# Comprehensive Molecular Profiling of Colorectal Cancer With Situs Inversus Totalis by Next-Generation Sequencing

**DOI:** 10.3389/fonc.2022.813253

**Published:** 2022-04-20

**Authors:** Hongsen Li, Liu Gong, Huanqing Cheng, Huina Wang, Xiaochen Zhang, Chuangzhou Rao, Zhangfa Song, Da Wang, Haizhou Lou, Feng Lou, Shanbo Cao, Hongming Pan, Yong Fang

**Affiliations:** ^1^ Department of Medical Oncology, Sir Run Run Shaw Hospital, Zhejiang University School of Medicine, Hangzhou, China; ^2^ Prenatal Diagnosis Center, Affiliated Hospital of Weifang Medical University, Weifang, China; ^3^ Acornmed Biotechnology Co., Ltd., Beijing, China; ^4^ Department of Medical Oncology, The First Affiliated Hospital, Zhejiang University School of Medicine, Hangzhou, China; ^5^ Department of Radiotherapy and Chemotherapy, Hwamei Hospital, University of Chinese Academy of Sciences, Ningbo, China; ^6^ Department of Anorectal Surgical, Sir Run Run Shaw Hospital, Zhejiang University School of Medicine, Hangzhou, China

**Keywords:** colorectal cancer, SCRC, NSCRC, mutational profile, next-generation sequencing

## Abstract

**Background:**

Colorectal cancer (CRC) is one of the most prevalent malignances worldwide. However, CRC with situs inversus totalis (SCRC) is extremely rare, and molecular characterization of this disease has never been investigated.

**Methods:**

Tumor tissue samples from 8 patients with SCRC and 33 CRC patients without situs inversus totalis (NSCRC) were subjected to multigene next-generation sequencing.

**Results:**

The most frequently mutated genes in SCRC were *APC*, *TP53*, *CHEK2*, *MDC1*, *GNAQ*, *KRAS*, and *SMAD4*. A high frequency of SCRC tumors had mutations in DNA damage repair genes. Single amino acid substitutions in the DNA damage repair genes caused by continuous double base substitution was identified in the majority of this population. Furthermore, mutational profiles showed notable differences between the SCRC and NSCRC groups. In particular, *CHEK2*, *MDC1*, *GNAQ*, *SMAD4*, *BRCA1*, *HLA-B*, *LATS2*, and *NLRC5* mutations were more frequently observed in SCRC patients. The mutation loci distributions of *KRAS* in the SCRC cohort differed from that of the NSCRC cohort. Additionally, differences in the targeted genomic profiles and base substitution patterns were observed between the two groups.

**Conclusions:**

These findings comprehensively revealed a molecular characterization of SCRC, which will contribute to the development of personalized therapy and improved clinical management of SCRC patients.

## Introduction

Colorectal cancer (CRC) is the third most prevalent type of cancer and the second leading cause of cancer-associated mortality worldwide (1). In recent years, the incidence and mortality rates for CRC have been steadily increasing (2). CRC patients can achieve promising clinical outcomes with early diagnosis. However, most CRC patients are generally diagnosed at an advanced stage, and over 50% of CRC patients develop liver, lung and lymph node metastases with a high mortality rate (3).

A series of genomic mutations leads to the development and progression of CRC. In recent years, next-generation sequencing (NGS) has revealed a diversity of driver gene mutations and affected signaling pathways in CRC. Numerous studies have demonstrated that the most commonly mutated driver genes in CRC are *APC*, *TP53*, *KRAS*, *SMAD4*, and *PIK3CA* (4, 5). Additionally, the Cancer Genome Atlas network comprehensively investigated the mutational landscape and identified some novel genomic alterations in CRC (6). The WNT, TGF-β PI3K, RAS/MAPK, and p53 signaling pathways were found to be the most frequently altered pathways in CRC (6). Recurrent copy number variations included the amplification of *ERBB2* and *IGF2*, and frequent chromosomal rearrangements included the fusion of *NAV2* and *TCF7L1* (6). Multi-omic results classified the CRC patients into hypermutated (16%) and non-hypermutated (84%) groups, and the hypermutated subgroup was characterized by high microsatellite instability, hypermethylation, *MLH1* silencing, and genomic mutations in mismatch repair genes and *POLE* (6). Moreover, some large cohort studies found statistically significant differences in mutation status between right-sided and left-sided CRC for several genes, including *KRAS*, *BRAF*, and *FBXW7*, as well as for several pathways, including RAS/MAPK, TGF-β, and p53 signaling pathways (5, 7).

Situs inversus is an extremely rare congenital condition, with an overall incidence rate of 0.01% (8). The majority of situs inversus cases are situs inversus totalis, which are characterized by a left-to-right reversal of all the thoracic and abdominal organs. A growing number of people with situs inversus totalis have been identified because of advances in medical imaging technology. A large amount of literature has been published that covers treatments and appropriate operative approaches for CRC patients with situs inversus totalis (SCRC) (9–11). However, the molecular characterization of SCRC has been largely unexplored. In addition, genomic differences between SCRC patients and CRC patients without situs inversus totalis (NSCRC) are currently unknown.

To better understand SCRC disease biology and to elucidate the molecular features of SCRC, we systematically explored the molecular signatures in SCRC by NGS, and further compared these signatures between SCRC and NSCRC patients. These findings provide valuable genomic information for SCRC, which will contribute to promoting treatment strategies and clinical management of SCRC patients.

## Materials and Methods

### Patients and Sample Collection

A total of 41 patients with CRC from the Affiliated Sir Run Run Shaw Hospital of Zhejiang University School of Medicine and the First Affiliated Hospital of Zhejiang University School of Medicine between January 2014 and January 2020 were enrolled. Eight SCRC and 33 NSCRC patients were included. Clinical stages of patients ranged from I to IV, which was verified based on the American Joint Committee on Cancer staging scheme (8th edition). This study was approved by the ethical committee of the Affiliated Sir Run Run Shaw Hospital of Zhejiang University School of Medicine. Informed consent was obtained from all participants.

### NGS

Tissue DNA was extracted utilizing the QIAamp Genomic DNA kit (Germany, QIAGEN) based on the manufacturer’s instructions. Sequencing libraries were generated according to Illumina standard library construction protocols (Illumina Inc.). The libraries were enriched using an Acornmed panel targeting 808 cancer-related genes. The captured libraries were then sequenced on the NovaSeq6000 System (Illumina Inc.). Sequencing reads were aligned to the reference human genome (hg19) using the BWA aligner (version 0.7.12). Base recalibration was conducted using GATK software (version 3.8). Single nucleotide variants (SNVs) and small insertions or deletions were identified using MuTect2 software (version 1.1.7). CONTRA software (version 2.0.8) was used to analyze copy number variant calling. An average coverage depth for tumor tissue was > 5000×. Mutations were identified based on these standards: mutant allele frequency (MAF) ≥ 1.0%, and at least 5 high-quality supporting reads (base quality ≥ 30, mapping quality ≥ 30).

### Identification of Potentially Actionable Mutations With OncoKB

Potentially actionable mutations were identified using MSK’s Precision Oncology Knowledge Base (OncoKB) (https://www.oncokb.org/). This clinical support tool distills information from FDA approved regimens, National Comprehensive Cancer Network (NCCN) guidelines, and the published scientific literature. Clinically relevant alterations were classified into one of four levels according to the strength of evidence. Mutations are annotated by the level of evidence supporting the use of certain drugs. The levels of evidence are as follows:

Level 1: FDA-recognized biomarker predictive of response to an FDA-approved drug in this indication.
Level 2: Standard care biomarker recommended by the NCCN or other professional guidelines predictive of response to an FDA-approved drug in this indication.
Level 3A: Compelling clinical evidence supports the biomarker as being predictive of response to a drug in this indication.
Level 3B: Standard care or investigational biomarker predictive of response to an FDA-approved or investigational drug in another indication.
Level 4: Compelling biological evidence supports the biomarker as being predictive of response to a drug.

### Statistical Analysis

SPSS 21.0 statistical software (IBM Corp.) was used to conduct our statistical analyses. A Fisher’s exact test was used to determine the associations between gene mutation status and clinical characteristics. Differences in continuous variables were measured using a Student’s *t*-test. A two-sided *P*< 0.05 was considered statistically significant.

## Results

### Patient Features

In the present study, a total of eight SCRC patients and 33 NSCRC patients were included. Among the SCRC patients, the median age at diagnosis was 60 years old (range, 51-75 years old). Seventy-five percent of the patients were female, and none of them were smokers. In the NSCRC cohort, the median age at diagnosis was also 60 years old (range, 28-79 years old). A total of 36.4% of the patients were female, and 24.2% of them were smokers. We further compared the clinical characteristics between the SCRC and NSCRC cohorts. There was no difference in the age between the two groups. However, compared with NSCRC patients, SCRC patients were more likely to be female (*P* = 0.048). The clinical and pathological characteristics of the patients are listed in **Table 1**.

**Table 1:** Clinical characteristics of SCRC and NSCRC patients.

Characteristics	Total (n = 41)	SCRC (n = 8)	NSCRC (n = 33)	*P* value
Age, year, median (range)	60 (28-79)	60 (51-75)	60 (28-79)	0.651
Gender, n (%)				
Male	23 (56.1%)	2 (25.0%)	21 (63.6%)	0.048
Female	18 (43.9%)	6 (75.0%)	12 (36.4%)	
Smoking history, n (%)				
Yes	8 (19.5%)	0 (0)	8 (24.2%)	0.121
No	33 (80.5%)	8 (100.0%)	25 (75.8%)	
Clinical Stage, n (%)				
I	1 (2.4%)	0	1 (3.0%)	0.226
II	1 (2.4%)	0	1 (3.0%)	
III	19 (46.3%)	7 (87.5%)	12 (36.4%)	
IV	18 (44.0%)	0	18 (54.6%)	
Unknown	2 (4.9%)	1 (12.5%)	1 (3.0%)	

### Mutational Landscape in SCRC

**Figure 1:**
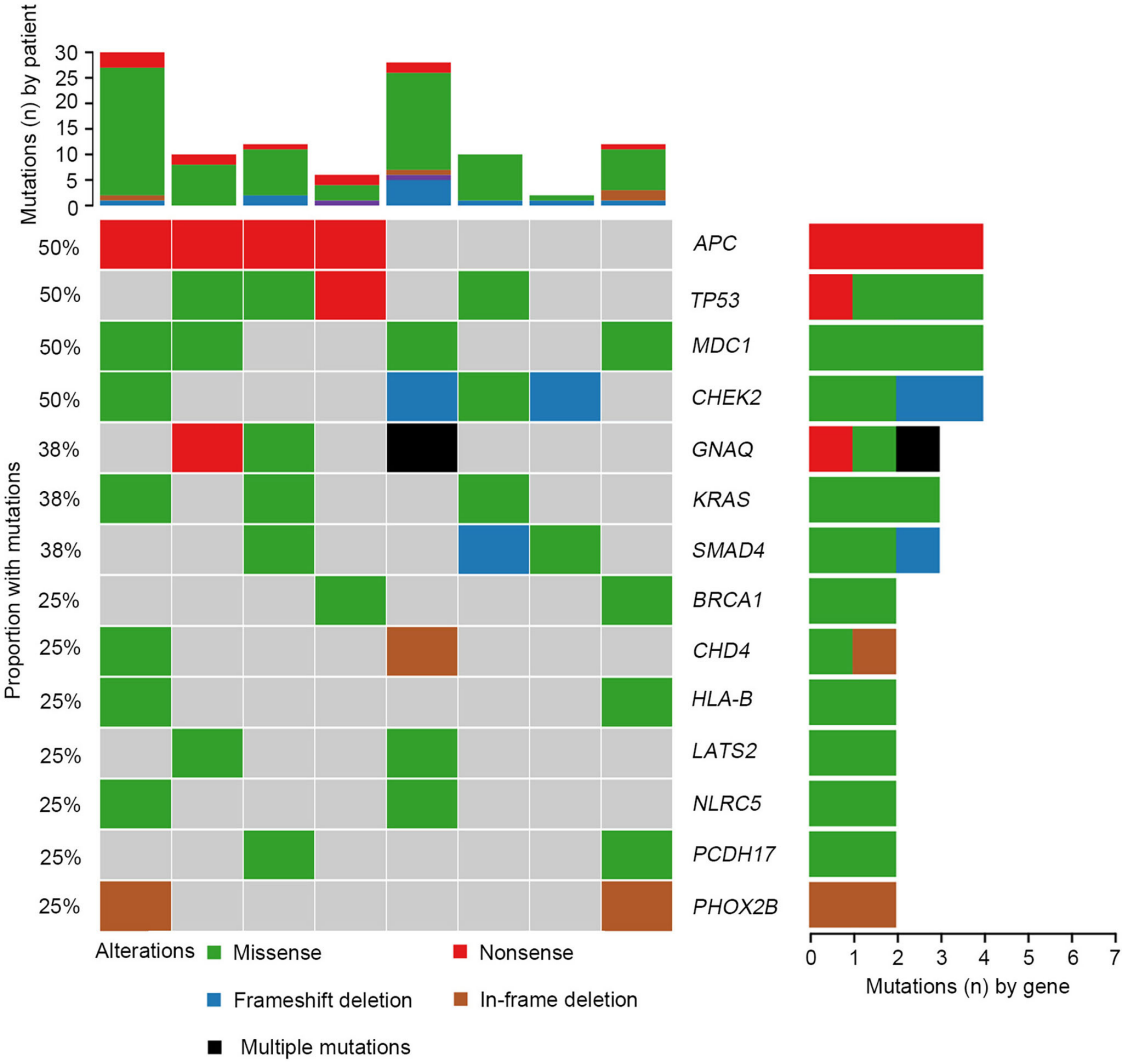
Mutational landscape of colorectal cancer patients with situs inversus totalis (SCRC). Genomic mutations were identified by targeted NGS of the tumor tissues from patients with SCRC. Multiple mutations, mutations numbers greater than two.

Samples from SCRC patients were profiled by targeted sequencing with an 808 cancer-related gene panel. An average coverage depth > 5000× was obtained for tumor tissue samples. The quality criteria used for mutation identification were a detection threshold of 1.0% and at least 5 high-quality supporting reads. All sequenced samples had at least one genetic alteration. A total of 110 somatic variants from 75 genes were identified. The most commonly mutated genes were *APC* (50%), *TP53* (50%), *CHEK2* (50%), *MDC1* (50%), *GNAQ* (38%), *KRAS* (38%), and *SMAD4* (38%) (**Figure 1**). Copy number variations in *ERBB2*, *MRE11A*, *FANCM*, *BRD4*, and *TOP1* were found (**Table S1**). Gene rearrangements were identified in one SCRC patient including *NTRK1-TPM3*. Among the *KRAS* mutations, only *G13D* substitutions were observed.

**Figure 2:**
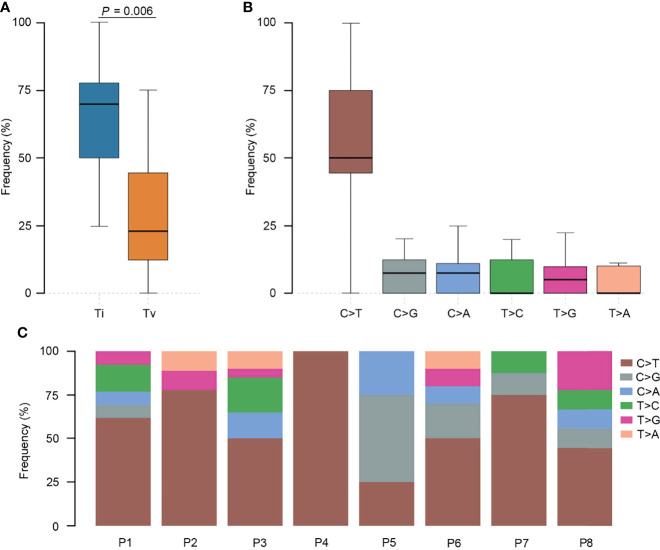
Transition (Ti) and transversion (Tv) profiles for SCRC. **(A)** Comparison between Ti and Tv ratios in SCRC. **(B)** The bar plot shows each type of Ti or Tv. **(C)** The box plot exhibits the ratio of each type of Ti or Tv for each patient.

We further investigated the affected signaling pathways in SCRC based on the mutation data. The DNA damage repair (DDR), WNT, RAS/MAPK, TGF-β, and p53 signaling pathways were the most commonly altered pathways. Additionally, the Ca^2+^, epigenetic, IFN-γ, NOTCH, and PI3K signaling pathways were affected in some patients (**Table S2, S3**). Notably, the DDR pathway was strikingly altered in SCRC (87.5%) patients, and the majority of these patients (71.4%) had a single amino acid substitution in the DDR genes caused by continuous double base substitution (**Table 2**). To further understand the background of carcinogenesis, the pattern of nucleotide substitution was explored. All SNVs could be classified into transition (Ti) and transversion (Tv) substitutions according to the specific substitutions of pyrimidines and purines. The profile of base substitutions in SCRC showed the frequency of Ti was significantly higher than that of Tv (*P* = 0.006) (**Figure 2A**). Additionally, a high frequency of C > T Ti at the CpG dinucleotide was identified in 58.7% of SNVs (**Figures 2B, C**).

**Table 2:** Single amino acid substitutions of DDR genes caused by continuous double base substitution.

Patient No.	DDR genes	Exon	Mutations
P1	*CHEK2*	Exon 11	c.1116_1117TG>CA; p.K416E
P1	*NLRC5*	Exon 13	c.2570_2571AC>GT; p.H857R
P3	*NLRC5*	Exon 13	c.2570_2571AC>GT; p.H857R
P5	*BRCA1*	Exon 13	c.4369_4370TG>CA; p.H1457C
P7	*CHEK2*	Exon 11	c.1116_1117TG>CA; p.K416E
P8	*MDC1*	Exon 10	c.3909_3910TG>CA; p.K1304E

### Comparison of Genomic Profiles Between SCRC and NSCRC Patients

**Figure 3:**
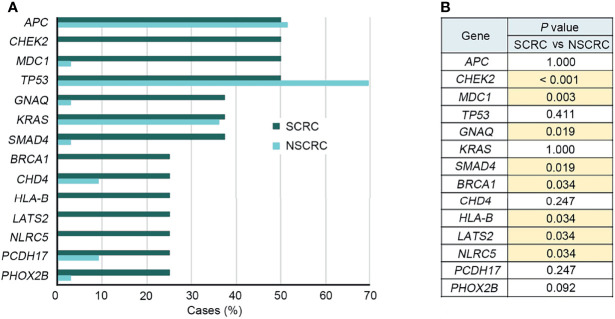
Comparison of the genomic landscape between SCRC and NSCRC cohorts. **(A)** Frequencies of genomic mutations in SCRC and NSCRC cohorts. **(B)** Corresponding *P* values comparing the prevalence of a gene’s mutations between different cohorts. Cases with a statistically significant difference (*P* < 0.05) are highlighted in yellow.

**Figure 4:**
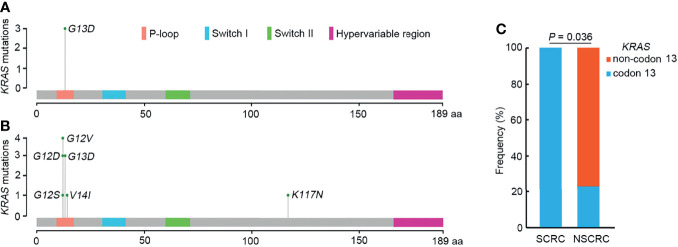
The mutation loci distributions of *KRAS* in SCRC and NSCRC groups. **(A)** Distributions of *KRAS* mutations identified in SCRC. **(B)** Distribution of *KRAS* mutations identified in NSCRC. **(C)** Comparison of the distributions of *KRAS* mutations between SCRC and NSCRC patients.

**Figure 5:**
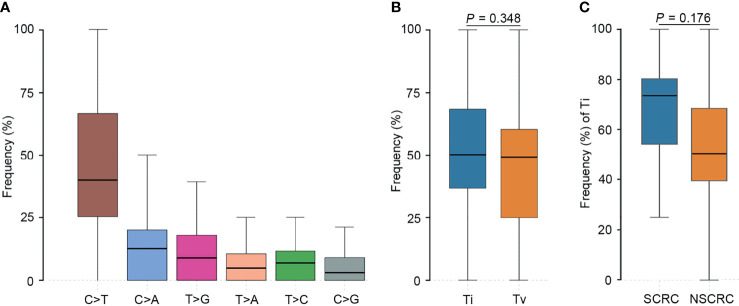
Comparison of the profiles of transition (Ti) and transversion (Tv) between SCRC and NSCRC patients. **(A)** The bar plot shows each type of Ti or Tv in NSCRC. **(B)** Comparison between Ti and Tv ratios in NSCRC. **(C)** Comparison of Ti ratios between the SCRC and NSCRC groups.

To further elucidate the molecular basis of SCRC tumorigenesis, a comprehensive comparison of genomic profiles between SCRC and NSCRC was conducted. Compared with NSCRC patients, significantly more genomic alterations were observed in *CHEK2*, *MDC1*, *GNAQ*, *SMAD4*, *BRCA1*, *HLA-B*, *LATS2*, and *NLRC5* among SCRC patients. A potential statistical difference was identified in *PHOX2B* between the two group (*P* = 0.092) (**Figures 3A, B**). The incidence of *KRAS* mutations between the two groups was not significantly different (**Figures 3A, B**). The mutation loci distribution of *KRAS* was further evaluated. In the SCRC group, *KRAS* mutations were only distributed on codon 13 (**Figure 4A**). However, in the NSCRC group, *KRAS* mutations were distributed on different codons (**Figure 4B**). Further analysis indicated that the frequencies of mutation loci distributions of *KRAS* between the SCRC and NSCRC groups showed a statistically significant difference (*P* = 0.036) (**Figure 4C**). Moreover, the profile of base substitutions between the two groups was found to be different. A total of 34.7% of SNVs identified were C > T Ti in NSCRC (**Figure 5A**), which was lower than that in SCRC (34.7% vs 58.7%). No difference between the frequencies of Ti and Tv in NSCRC was observed (**Figure 5B**). The proportion of Ti in SCRC tended to be higher than that in NSCRC, although no statistically significant difference was observed (**Figure 5C**).

**Figure 6:**
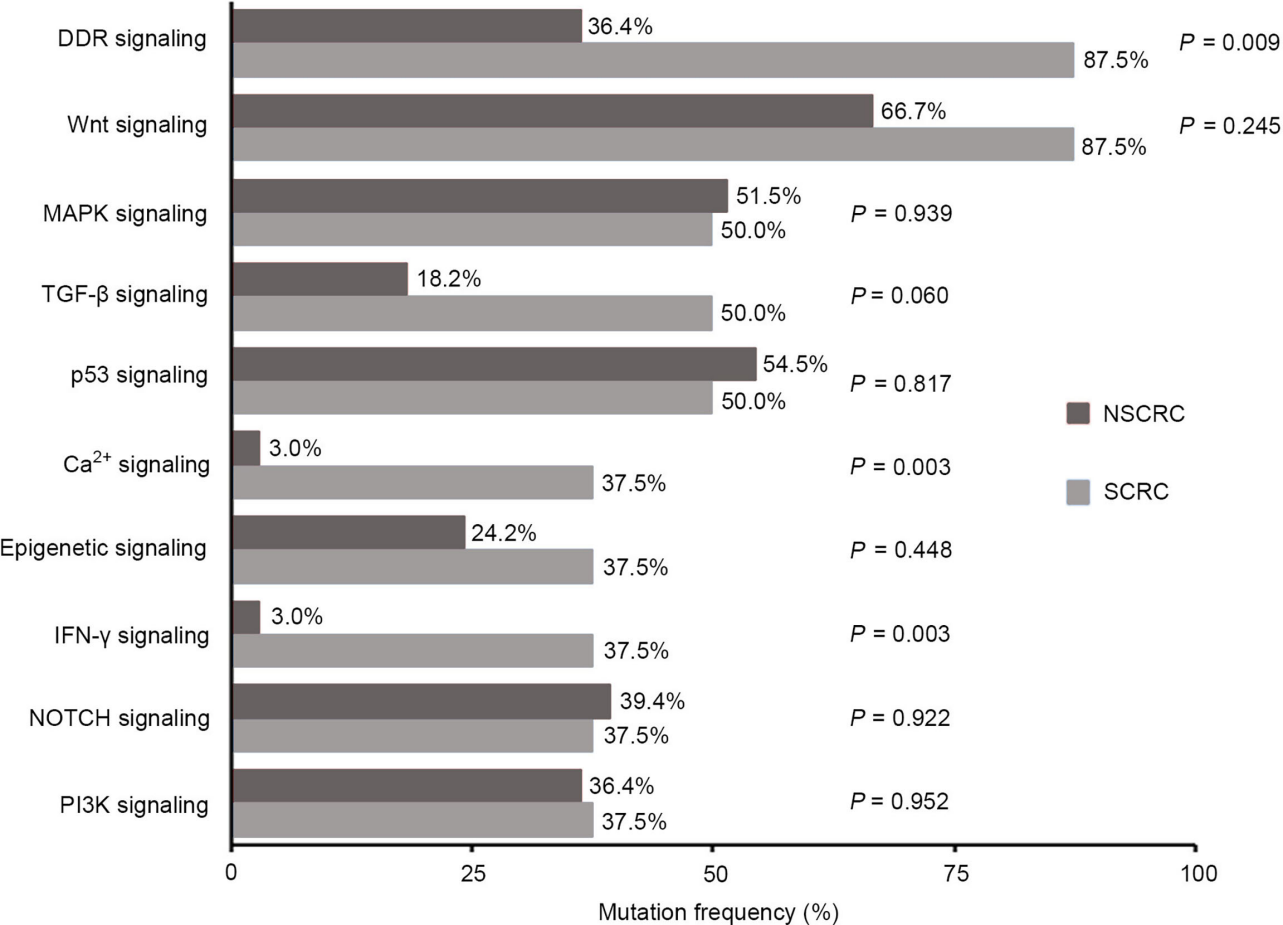
Comparison between SCRC and NSCRC patients of the detection rate of genomic mutations in signaling pathways.

**Figure 7:**
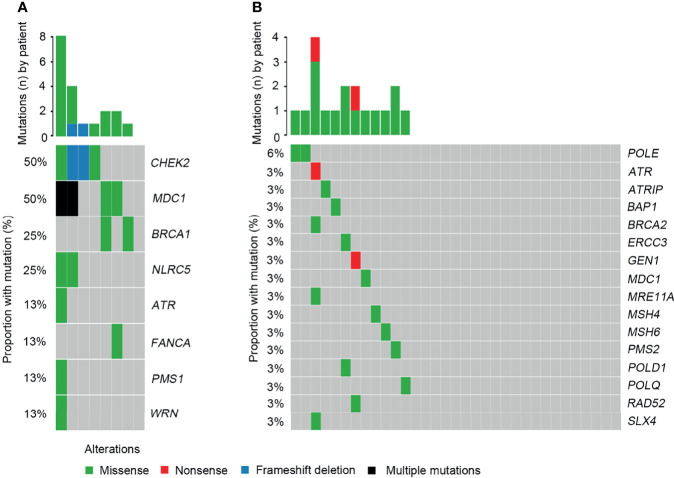
Comparison of the DDR profiles between SCRC and NSCRC patients. **(A)** The DDR profile in the SCRC group. **(B)** The DDR profile in the NSCRC group.

When investigating differences in altered signaling pathways, we found that the frequencies of the DDR, Ca^2+^, and IFN-γ signaling pathways in SCRC patients were significantly higher than those in NSCRC patients. A statistical trend was observed for the TGF-β pathway between the two cohorts (*P* = 0.060)(**Figure 6**). Notably, the mutated genes in the DDR signaling pathway between the two cohorts were markedly different. In SCRC, the most commonly altered DDR genes were *CHEK2* (50%), *MDC1* (50%), *BRCA1* (25%), and *NLRC5* (25%) (**Figure 7A**). However, the most frequently mutated DDR gene in NSCRC was *POLE* (6%). The other DDR mutations occurred in only one patient (**Figure 7B**). Furthermore, the phenomenon of single amino acid variations in DDR genes caused by continuous double base substitutions was not detected in NSCRC.

**Figure 8:**
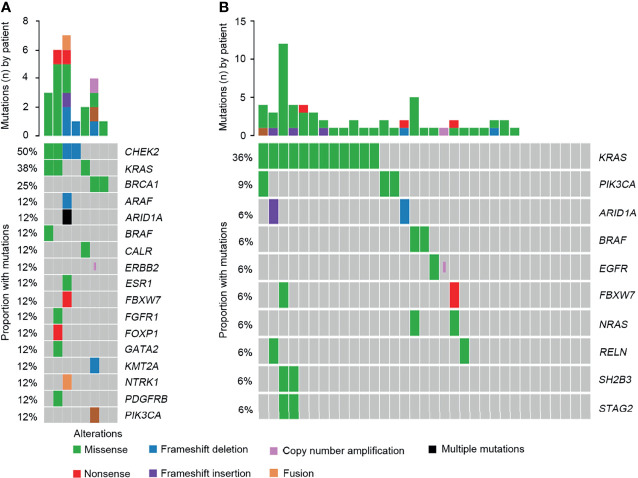
Comparison of the targeted genomic profiles between CRC and NSCRC patients. **(A)** The targeted genomic profile in the SCRC group. **(B)** The targeted genomic profile in the NSCRC group.

According to the OncoKB classification system, the profile of clinically relevant alterations was comprehensively evaluated. Overall, 24 clinically relevant mutations were detected in 87.5% of SCRC patients. Alterations in *CHEK2*, *KRAS*, and *BRCA1* were the most common targets (**Figure 8A** and **Table S4**). One patient harboring a *BRAF Y633C* mutation was identified. Additionally, a clinically significant *NTRK1-TPM3* fusion was identified. Although prior large cohort studies have reported the distributions of NTRK rearrangements in various tumors (12), they had not been uncovered in SCRC. Additionally, 78.8% of NSCRC cases harbored at least one clinically relevant alteration. However, the most frequently identified mutations occurred in *KRAS* and *PIK3CA* for this group (**Figure 8B** and **Table S4**). *BRAF* alterations were observed in two patients, including *V600E* and *P655A* mutations.

## Discussion

Given the rarity of SCRC, the clinical characteristics and molecular features of SCRC remain largely unknown. Numerous studies indicated that CRC is more common in men than in women (13, 14). However, our study indicated that SCRC patients were more likely to be female, demonstrating that female gender has a larger impact on the prevalence of SCRC. With the advent of high-throughput sequencing technology, efforts have been made to identify the molecular characterization of CRC. However, most of the studies focused on CRC patients with normally positioned structures of the abdominal and thoracic cavities. No reports have described the molecular features of SCRC. For the first time, we investigated the molecular signatures of SCRC, which will be of great significance in understanding the biological characteristics of cancer cells and in promoting the clinical management of this population.

In the present study, we identified a large number of genes with point mutations, insertions, deletions, and copy number variations in SCRC. The most frequently mutated genes were *APC*, *TP53*, *CHEK2*, *MDC1*, *GNAQ*, *KRAS*, and *SMAD4*. It is known that *APC*, *TP53*, *KRAS*, and *SMAD4* were the common driver genes in CRC (4, 5). In addition to these genes, our analysis identified novel driver genes in SCRC, including *GNAQ*, *CHEK2*, and *MDC1*. *GNAQ* encodes heterotrimeric G protein alpha subunits, which are crucial for G protein-coupled receptor signaling. Numerous studies have demonstrated that *GNAQ* mutations can lead to constitutive activation of the downstream RAS/MAPK pathway (15, 16), further indicating the important role of the RAS/MAPK pathway in SCRC. *CHEK2* encodes a cell cycle checkpoint kinase involved in the DDR process (17). *MDC1* encodes a scaffold protein that functions as a platform for the recruitment of different DDR factors, such as *RNF8* and *53BP1*, to regulate the DDR process (18). Furthermore, we found that 87.5% of SCRC patients carried genomic mutations in DDR genes. The DDR system is mainly involved in the mismatch repair of both DNA single-strand breaks (SSB) and double-strand breaks (DSB) (19). It is necessary for genomic integrity, and alterations in DDR genes frequently result in DDR deficiency. Recently, Mok *et al.* revealed a higher risk of colorectal cancer in *BRCA1* mutation carriers through systematic review and meta-analysis (20). In clinical practice, many clinical trials have demonstrated *BRCA1* mutation was used to predict the treatment benefit from Poly (ADP-ribose) polymerase inhibitors (PARPi) (21, 22). *CHEK2* is one of the DDR genes which are frequently analyzed because of their important role on maintaining genomic stability. *CHEK2* mutation is widely reported in sporadic colorectal cancer and hereditary colorectal cancer (23–25). However, the prevalence of *CHEK2* mutation is relatively low in colorectal cancer (25–28). In this study, the frequency of *CHEK2* mutation in SCRC is 50%, whereas no *CHEK2* mutation is found in NSCRC. These findings suggest that DDR deficiency plays an important role in the development of SCRC. Notably, single amino acid substitutions in the DDR genes caused by continuous double base substitutions in SCRC were a novel observation. Although the mechanism of this observation is unknown, the role and clinical significance in SCRC should be further explored.

Previous studies have reported that genomic mutations in CRC are dominated by C to T Ti at CpG sites, indicating that the deamination of 5-methylcytosine is a key initiating event in cancer-driving mutations (29). In our study, we found this phenomenon in SCRC and NSCRC simultaneously, whereas the ratio of C to T Ti in SCRC was higher than that in NSCRC. Additionally, the frequency of Ti in SCRC was significantly higher than that for Tv, but no difference was found between the frequencies of Ti and Tv in NSCRC. Furthermore, we comprehensively investigated the mutational landscape between SCRC and NSCRC and found that several genes had remarkably different mutation frequencies, including *CHEK2*, *MDC1*, *GNAQ*, *SMAD4*, *BRCA1*, *HLA-B*, *LATS2*, and *NLRC5*. In this study, targetable mutations, defined as molecular targets for drugs that can guide treatment decisions (30, 31), were analyzed. We found 87.5% of SCRC patients carried at least one clinically actionable mutation, and the difference was observed in the targetable mutation profile between SCRC and NSCRC patients. Thus, although there was a similarity between the mutational profiles of SCRC and NSCRC, SCRC still displayed a diverse mutational pattern. According to the mutation loci distributions of *KRAS*, it was found that patients with SCRC carried *KRAS* codon 13 mutations, whereas the majority (61.5%) of NSCRC patients harbored *KRAS* codon 12 mutations. In CRC, numerous studies revealed that *KRAS* codon 13 mutations displayed less tumorigenic activity than codon 12 mutations (32, 33). Moreover, experimental data demonstrated that *KRAS* codon 12 mutated tumors were more aggressive than codon 13-mutated tumors (34). A recent study by Tahir *et al.* exhibited that alterations at codon 12 and codon 13 in *KRAS* could lead to varying metastatic efficiencies and oncogenic transformation in colorectal cancer cell lines (35). In SCRC, only *KRAS* codon 13 mutations were found, which might lead to low tumorigenic activity in SCRC compared with NSCRC. Additionally, many studies have reported that *KRAS* codon 12 mutations are associated with a poor prognosis in patients with CRC (36, 37). The prognosis between the two groups may be different because of the differences in mutation loci distributions of *KRAS*. Therefore, it is critical to systematically investigate and understand the mutational spectrum of SCRC. Future treatments and clinical strategies for SCRC should be based on the specific molecular features.

In recent years, NGS has improved the understanding of the signal transduction cascades during the development and progression of CRC. It is known that the RAS/MAPK, PI3K, Wnt, and p53 signaling pathways are most affected in CRC (6). Besides these signaling pathways, we further revealed that Ca^2+^ and IFN-γ pathways were frequently affected in SCRC. Although numerous studies have demonstrated that the Ca^2+^ pathway plays an important role in the development and progression of many cancer types (38, 39), few reports revealed the role in CRC. This could be because of the low incidence of Ca^2+^ pathway alterations in CRC. In this study, the detection rate of Ca^2+^ pathway related gene alterations was significantly higher in SCRC compared with that in NSCRC, indicating a crucial role of this pathway in the development of SCRC. Recently, interferons, especially IFN-γ, have been found to act as key regulators in tumor immunotherapy (40). Interferons are mainly generated in response to immune stimuli or inflammation, and are crucial in tumor immunosurveillance (41). In SCRC, the common alterations in the IFN-γ pathway highlight the importance of understanding the interferon response, which may contribute to improving the design of immunotherapy trials in this population. Additionally, the DDR pathway is commonly affected in SCRC and NSCRC simultaneously, however, the detection rate of DDR alterations in SCRC is significantly higher than that in NSCRC (87.5% vs 37.5%). Furthermore, DDR gene alterations were remarkably different between the two groups. Recently, DDR deficiencies have become a novel predictive factor of response to immunotherapies, and mutations in DDR genes increases immunogenicity by enhancing the tumor neoantigen load (42). A number of studies have revealed that patients with DDR alterations benefit from PD-1/PD-L1 blockade in advanced urothelial and non-small cell lung cancer (43, 44). Therefore, the high mutation frequencies of genes related to the IFN-γ and DDR signaling pathways imply that immunotherapies may be an important treatment strategy in SCRC.

To the best of our knowledge, this is the first and largest study to characterize the mutational profile in SCRC, and in addition, to compare these profiles between SCRC and NSCRC patients. We revealed a diverse genomic landscape between the two groups as well as novel mutations for targets for future therapy in SCRC. Additionally, we discovered a feature of single amino acid substitutions in DDR genes caused by continuous double base substitutions, which was the first to be reported for CRC and other cancers. These results suggest that SCRC patients present with some unique molecular characteristics. These observations will contribute to promoting the development of personalized therapy and the clinical management in this population.

## Data Availability Statement

The sequencing data are available in the NGDC, GSA database (https://bigd.big.ac.cn/gsa/) under the accession number of PRJCA008881.

## Ethics Statement

The studies involving human participants were reviewed and approved by Ethical Committee of the Affiliated Sir Run Run Shaw Hospital of Zhejiang University School of Medicine. The patients/participants provided their written informed consent to participate in this study.

## Author Contributions

YF, HP, and SC designed the study. HZL, LG, XZ, CR, ZS, DW, and HSL enrolled the patients and collected the corresponding clinical information. HC, HW, and FL conducted the data analysis. HZL, HSL LG, HC, and HW wrote the paper. All authors contributed to the article and approved the final version.

## Funding

This work was supported by the National Natural Science Foundation of China (No. 81702809 and No. 81872238) and the Medical Science and Technology Project of Zhejiang Province (No. 2016ZDB007 and No. 2017ZD021).

## Conflict of Interest

HW, FL, and SC are employees of Acornmed Biotechnology Co., Ltd.

The remaining authors declare that the research was conducted in the absence of any commercial or financial relationships that could be construed as a potential conflict of interest.

## Publisher’s Note

All claims expressed in this article are solely those of the authors and do not necessarily represent those of their affiliated organizations, or those of the publisher, the editors and the reviewers. Any product that may be evaluated in this article, or claim that may be made by its manufacturer, is not guaranteed or endorsed by the publisher.
