# Supplementary material for: Comprehensive Molecular Profiling of Colorectal Cancer With Situs Inversus Totalis by Next-Generation Sequencing
Source: Front Oncol. 2022 Apr 20;12:813253. doi: 10.3389/fonc.2022.813253 (PMC9067615; doi:10.3389/fonc.2022.813253)
Supplement: Supplementary file 1 [file Table_1.docx]

Supplementary Material

# Supplementary Tables

**Table S1.** Copy number variations identified in SCRC.

| Patient No. | Copy number variations |
| --- | --- |
| P1 | *BRD4* gain |
| P2 | *ERBB2* gain |
| P2 | *MRE11A* loss |
| P2 | *FANCM* loss |
| P5 | *TOP1* gain |
| P5 | *MRE11A* loss |
| P5 | *FANCM* loss |

**Table S2.** Frequencies of commonly mutated pathways in SCRC.

| Signaling pathways | No. of tumors | Proportion (%) |
| --- | --- | --- |
| DNA damage repair | 7 | 87.5% |
| WNT | 7 | 87.5% |
| MAPK | 4 | 50.0% |
| TGF-β | 4 | 50.0% |
| p53 | 4 | 50.0% |
| Ca^2+^ | 3 | 37.5% |
| Epigenetic | 3 | 37.5% |
| IFN-γ | 3 | 37.5% |
| NOTCH | 3 | 37.5% |
| PI3K/AKT | 3 | 37.5% |

**Table S3.** The pathways and genes involved in the 808-cancer-gene panel.

| Signaling pathways | Genes involved |
| --- | --- |
| DNA damage repair | *ATM, ATR, ATRIP, BAP1, BLM, BRCA1, BRCA2, BRIP1, CHEK1, CHEK2, CUL3, CUL4A, DCLRE1C, DDB1, ERCC1, ERCC2, ERCC3, ERCC4, ERCC5, FANCA, FANCB, FANCC, FANCD2, FANCE, FANCF, FANCG, FANCI, FANCL, FANCM, GEN1, LIG3, LIG4, MBD4, MDC1, MLH1, MLH3, MNAT1, MRE11A, MSH2, MSH3, MSH6, MUTYH, NBN, NEIL1, NEIL3, NTHL1, PALB2, PARP1, PARP2, PARP3, PARP4, PER1, PMS1, PMS2, POLD1,POLE, POLM, POLQ, PPP4R2, PRKDC, RAD50, RAD51, RAD51B, RAD51C, RAD51D, RAD52, RAD54B, RAD54L, RECQL, RECQL4, REV1, REV3L, RPA1, SLX4, TDG, USP1, WRN, XRCC1, XRCC2, XRCC3* |
| Wnt/β-catenin | *AMER1, APC, AXIN1, AXIN2, BCOR, CDC42, CDC73, CFTR, CHD2, CHD4, CTNNA1, CTNNA2, CTNNB1, FH, GSK3B, KIF2B, LRP1B, LZTR1, NUTM1, PAK6, PCDH10, PCDH17, RNF43, SALL1, SOX17, SOX2, SOX9, TCF7L2* |
| MAPK | *ARAF, BRAF, BRD4, CREBBP, ETV1, HRAS, HSP90AA1, KRAS, MAP2K1, MAP2K2, MAP2K4, MAP3K1, MAP3K13, MST1R, NF1, NRAS, RAF1, RIT1* |
| TGF-β | *ACVR1, ACVR1B, ACVR2A, ENG, INHBA, SMAD2, SMAD3, SMAD4, TGFBR1, TGFBR2, ZNF217, ZNF521, ZNF536, ZNF804A, ZNF804B, ZNF831* |
| p53 | *ADGRA2, ALOX12B, MDM2, MDM4, PXDNL, RPS6KA4, TP53, TP53BP1* |
| Ca^2+^ | *SLC8A1, GNAQ, GABRA2, CHRM2* |
| Epigenetic | *ASXL1, ATRX, AURKB, BCORL1, DNAJB1, DNMT1, DNMT3A, DNMT3B, EP300, H3F3A, HFE, HIST1H2BD, HIST1H3B, HIST1H3C, HIST1H3D, HIST1H3J, KAT6A, KDM5C, KDM6A, KMT2A, KMT2B, KMT2C, KMT2D, KMT5A, NCOA3, NSD1, PBRM1, PRDM1, RPS24, SETBP1, SETD2, SF3B1, SMARCA4, SMARCD1, TAF1, TOP2A, TRIM58* |
| IFN-γ | *ARID2, B2M, HLA-A, NLRC5* |
| NOTCH | *NOTCH1, NOTCH2, NOTCH3, NOTCH4, KDM5A, SPEN, FBXW7, NCOR1, EGFL7, TMPRSS2* |
| PI3K/AKT | *AKT1, AKT2, AKT3, DROSHA, EIF4A2, EPHA3, FAM135B, GABRA6, INPP4A, INPP4B, INPPL1, MAPKAP, MTOR, PDK1, PIK3C2B, PIK3C2G, PIK3C3, PIK3CA, PIK3CB, PIK3CD, PIK3CG, PIK3R1, PIK3R2, PIK3R3, PPP2R1A, PREX2, PRKACA, PRKAR1A, PTEN, RHEB, RICTOR, RPTOR, STK11, TSC1, TSC2, UPF1, WT1* |

**Table S4.** Targetable alterations in SCRC and NSCRC groups.

| **Group** | **Patient number** | **Targetable alterations** |
| --- | --- | --- |
| SCRC | P1 | CHEK2 p.K416E |
| SCRC | P1 | FGFR1 p.H1457C |
| SCRC | P1 | FOXP1 p.K416E |
| SCRC | P1 | GATA2 p.K1304E |
| SCRC | P1 | KRAS p.G13D |
| SCRC | P1 | PDGFRB p.V779I |
| SCRC | P2 | BRCA1 p.T922I |
| SCRC | P2 | KMT2A p.T2727fs |
| SCRC | P2 | PIK3CA p.12_18del |
| SCRC | P2 | ERBB2 copy number gain |
| SCRC | P3 | ARAF p.T253fs |
| SCRC | P3 | ARID1A p.Y1226C |
| SCRC | P3 | ARID1A p.G1847fs |
| SCRC | P3 | CHEK2 p.P388fs |
| SCRC | P3 | ESR1 p.V51M |
| SCRC | P3 | FBXW7 p.E452X |
| SCRC | P3 | NTRK1-TPM3 fusion |
| SCRC | P4 | CHEK2 p.G403fs |
| SCRC | P5 | BRCA1 p.H1457C |
| SCRC | P6 | CALR p.P301A |
| SCRC | P6 | KRAS p.G13D |
| SCRC | P7 | BRAF p.Y633C |
| SCRC | P7 | CHEK2 p.K416E |
| SCRC | P7 | KRAS p.G13D |
| NSCRC | P1 | PIK3CA p.Q546K |
| NSCRC | P2 | KRAS p.G13D |
| NSCRC | P3 | CREBBP p.Q2199L |
| NSCRC | P4 | RELN p.T2389I |
| NSCRC | P5 | KRAS p.G12S |
| NSCRC | P5 | PPM1D p.Q510fs |
| NSCRC | P6 | KDM6A p.K1095fs |
| NSCRC | P6 | STAT3 p.D661Y |
| NSCRC | P7 | BRAF p.P655A |
| NSCRC | P7 | ETV6 p.S257R |
| NSCRC | P7 | MTOR p.V21A |
| NSCRC | P7 | NRAS p.Q61R |
| NSCRC | P7 | TYK2 p.I960M |
| NSCRC | P8 | ARID1A p.T2150fs |
| NSCRC | P8 | ASXL1 p.Y591_Q592delinsX |
| NSCRC | P9 | FBXW7 p.R222X |
| NSCRC | P9 | NRAS p.G12D |
| NSCRC | P10 | KRAS p.G12D |
| NSCRC | P11 | BRCA2 p.P845L |
| NSCRC | P11 | DNMT3A p.R326H |
| NSCRC | P11 | FBXW7 p.T53N |
| NSCRC | P11 | JAK3 p.A60T |
| NSCRC | P11 | KRAS p.G12D |
| NSCRC | P11 | MALT1 p.S674P |
| NSCRC | P11 | SH2B3 p.R73C |
| NSCRC | P11 | SOCS1 p.M1I |
| NSCRC | P11 | STAG2 p.R69Q |
| NSCRC | P11 | STAT5B p.A693S |
| NSCRC | P11 | TCF3 p.H387Q |
| NSCRC | P11 | TSC1 p.M18I |
| NSCRC | P12 | KRAS p.G12V |
| NSCRC | P12 | NF1 p.L1715fs |
| NSCRC | P12 | SH2B3 p.A21V |
| NSCRC | P12 | STAG2 p.A214G |
| NSCRC | P13 | EGFR p.E391K |
| NSCRC | P14 | ERBB2 p.D384Y |
| NSCRC | P14 | PIK3CA p.S514R |
| NSCRC | P15 | JAK3 copy number gain |
| NSCRC | P16 | ARID1A p.S2249fs |
| NSCRC | P16 | KRAS p.G12V |
| NSCRC | P16 | RELN p.E1074G |
| NSCRC | P17 | EGFR copy number gain |
| NSCRC | P18 | KMT2D p.M3592L |
| NSCRC | P18 | KMT2D p.K2032Q |
| NSCRC | P19 | KRAS p.G12D |
| NSCRC | P19 | PIK3CA p.R108H |
| NSCRC | P19 | PIK3CA p.E39K |
| NSCRC | P19 | PTEN p.273_276del |
| NSCRC | P20 | KRAS p.G12V |
| NSCRC | P20 | KRAS p.V14I |
| NSCRC | P21 | KRAS p.K117N |
| NSCRC | P22 | KRAS p.G13D |
| NSCRC | P23 | ABL2 p.V776M |
| NSCRC | P23 | CDK12 p.R902X |
| NSCRC | P23 | FGFR3 p.K404R |
| NSCRC | P23 | KRAS p.G12V |
| NSCRC | P24 | SETBP1 p.Q108H |
| NSCRC | P25 | BRAF p.V600E |
| NSCRC | P26 | FGFR1 p.E126D |
| NSCRC | P26 | HRAS p.G12C |
| NSCRC | P26 | KRAS p.G13D |
